# Selected Flavonoids to Target Melanoma: A Perspective in Nanoengineering Delivery Systems

**DOI:** 10.3390/bioengineering9070290

**Published:** 2022-06-29

**Authors:** Tiago E. Coutinho, Eliana B. Souto, Amélia M. Silva

**Affiliations:** 1 Center for Research and Technology of Agro-Environmental and Biological Sciences (CITAB-UTAD), University of Trás-os-Montes e Alto Douro (UTAD), Quinta de Prados, 5001-801 Vila Real, Portugal; tecoutinho@utad.pt; 2 Department of Biology and Environment, School of Life Sciences and Environment, UTAD, Quinta de Prados, 5001-801 Vila Real, Portugal; 3 Department of Pharmaceutical Technology, Faculty of Pharmacy, University of Porto, Rua de Jorge Viterbo Ferreira, 228, 4050-313 Porto, Portugal; ebsouto@ff.up.pt; 4 UCIBIO/REQUIMTE, Faculty of Pharmacy, University of Porto, Rua de Jorge Viterbo Ferreira, 228, 4050-313 Porto, Portugal

**Keywords:** melanoma, apigenin, epigallocatechin-3-gallate, kaempferol, naringenin, silybin, nanoparticles

## Abstract

Melanoma is a complex type of cancer that depends on several metabolic factors, while the currently used therapies are not always effective and have unwanted side effects. In this review, the main factors involved in the etiology of cutaneous carcinoma are highlighted, together with the main genes and proteins that regulate cancer invasion and metastization. The role of five selected flavonoids, namely, apigenin, epigallocatechin-3-gallate, kaempferol, naringenin, and silybin, in the modulating receptor tyrosine kinase (RTK) and Wnt pathways is reported with their relevance in the future design of drugs to mitigate and/or treat melanoma. However, as phenolic compounds have some difficulties in reaching the target site, the encapsulation of these compounds in nanoparticles is a promising strategy to promote improved physicochemical stabilization of the bioactives and achieve greater bioavailability. Scientific evidence is given about the beneficial effects of loading these flavonoids into selected nanoparticles for further exploitation in the treatment of melanoma.

## 1. Introduction

**Figure 1:**
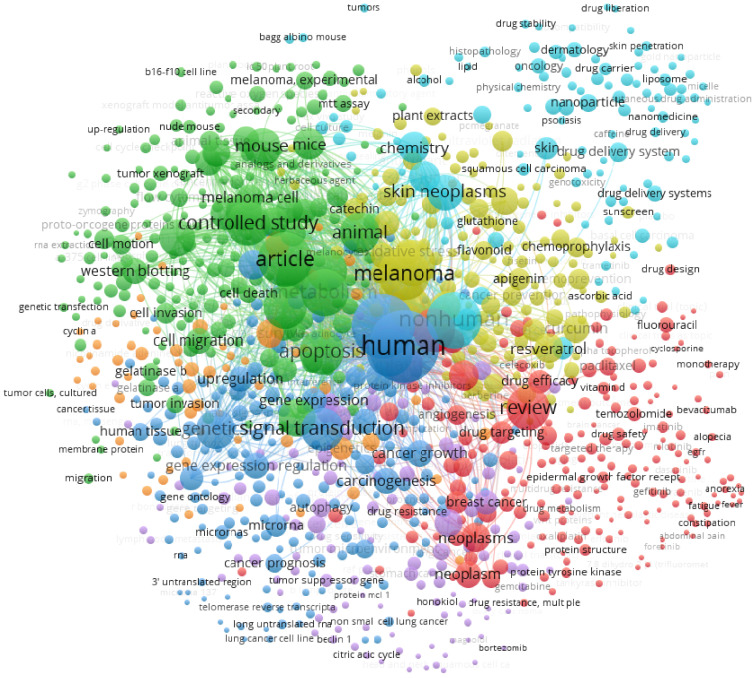
The bibliometric map obtained by VOSviewer software version 1.6.16 [18] using “cutaneous melanoma” AND “apigenin” OR “EGCG” OR “kaempferol” OR “naringenin” OR “Silybin” as keywords, recorded from the Scopus database.

Among the various types of cancer, melanoma has an increasing incidence worldwide annually, while representing ~1.8% of all cancers and less than 10% of skin cancers [1,2]. Despite its low incidence rates, it is considered as one of the most aggressive types of cancer, being responsible for approximately 80% of deaths in people with skin cancer due to the high invasion and metastasis capacity of the cancer cells [2,3]. The appearance of melanoma is attributed to several risk factors including environmental ones such as exposure to ultraviolet radiation (UVR) and host factors such as the number of dysplastic nevi, family history of disease, and genetic susceptibility [2,4]. An increased number of approved therapies for the treatment of melanoma has been seen over recent years, providing new alternative approaches that can be tailored to each specific case. The currently accepted alternatives range from chemotherapy to targeted therapy, immunotherapy, and combinations of those [5]. However, the heterogeneity of the type of melanoma, drug resistance, and the reduced quality of life of patients are still reported as major problems to overcome [6]. These can be mitigated by using phytochemicals that may contribute to reduce the side effects, and also through the synergic effect between phytochemicals and chemotherapeutic drugs, thereby increasing the effectiveness of the treatment while reducing some of the side effects. These phytochemicals can be polyphenols [7], which have high therapeutic potential, as they also have anticancer activities, beyond antioxidant and anti-inflammatory properties, and thus help in the control of physiological homeostasis [7,8,9]. Flavonoids (a class of polyphenolic compounds) are among the phytochemicals with the above-mentioned properties. Their central structure consists of three rings, two of them with six carbons and one of them with three carbons, in the form C6-C3-C6, being two aromatic phenyl rings and a heterocyclic ring. The difference in the subclass of flavonoids relies on the changes in this main structure. There are several subclasses of flavonoids such as flavones, flavanones, flavonols, anthocyanins, isoflavones, among others, which are distinguished by different unsaturation in the central ring, and substitutions throughout the central structure. These differences in structures allow for flavonoids to have several bioactivities of interest such as antioxidant, anti-inflammatory, antiviral, antifungal, and anticancer activity, among others [7,10,11,12,13]. Apigenin, epigallocatechin-3-gallate, kaempferol, naringenin, and silybin have high potential against melanoma and may, in the future, be used in the treatment (or as an adjuvant) of this condition [13,14,15,16]. However, there are several problems in the use of natural compounds for the treatment of melanoma such as their stability, the targeted-specific release at the site of action, and also their bioavailability at therapeutic concentrations, as the human body metabolizes these compounds by changing their structure as well as their activity [7,12]. To overcome these problems, the use of nanotechnology has been suggested. Nanoparticles have a high potential to entrap and carry the desired compounds, maintain their physico-chemical stability, increase their permeabilization, help them reach the target site, and even improve their activity [7,12,17]. Currently, nanoparticles are of the highest interest, despite several constraints that science seeks to solve, aiming to improve the quality of life of people who suffer from cancer. The current interest in using natural compounds such as flavonoids as anticancer agents or as metabolic protectors is evident by the vast number of articles published in the field. A search using the combination of terms “cutaneous melanoma” AND “apigenin” OR “EGCG” OR “kaempferol” OR “naringenin” OR “Silybin” resulted in 394 published papers in Scopus. To obtain the bibliometric map, the VOSviewer software was used (Figure 1) [18], resulting in seven clusters, highlighting a range of different studies on the use of the selected natural compounds for the treatment of cutaneous melanoma.

## 2. Cutaneous Melanoma

Skin cancers are usually split into two main types, melanoma and non-melanoma. UVR, which is mainly responsible for the development of melanoma, is highly modulated by melanin, and thus the skin phototype is a key epidemiologic risk factor with Caucasians at higher risk, and red-haired individuals showing about a three times higher risk than that observed in other Caucasians [19]. However, it was reported that Caucasians from Australia have about a 4–5 times higher risk of developing melanoma than Caucasian from Europe [1], thus the environment modulates the phototype risk factor such as the type of UVR, the solar incidence, and the time of exposure [20]. The individual’s genetics also play a crucial role such as the DNA repair genes, senescence, and pigmentation as well as melanoma cases in families [1,2,3].

Melanocytes have, as a primary function, the synthesis of melanin (melanogenesis), which is then transferred to the keratinocytes that surround the melanocytes. Melanogenesis is regulated by more than 125 different genes with the tyrosinase gene upregulated during melanogenesis, which is modulated by UVR exposure [21], and tyrosinase catalyzes the rate limiting reaction in melanin synthesis, which is the conversion of L-tyrosine into L-DOPA [1,22,23]. Although the primary role of melanin is to protect against UV-radiation, it is known that UVR exposure induces immunosuppression by decreasing the effectiveness of T- and NK (natural killer) cells (lymphocytes), DNA damage and creating mutations that encode the mutant proteins necessary in pathways responsible for cellular growth and differentiation such as the mitogen-activated protein kinase (MAPK), neuroblastoma rat sarcoma oncogene (NRAS), and the v-raf murine sarcoma viral oncogene homolog B (BRAF) [2,24]. UVR also has inflammatory properties, which consequently increase the formation of reactive oxygen species (ROS), also increasing the probability of developing DNA damage and mutations [25].

With respect to the host risk factor, it is known that about 10% of people with melanoma have a family history of the disease, and hereditary mutations are found in the *NRAS* gene, but they are mostly in the cyclin dependent kinase inhibitor 2A (*CDKN2A*) gene, which is involved in cell cycle regulation and progression as well as in senescence [2,25]. The factors above-mentioned lead to the initial alterations in melanocytes, leading to the formation of melanoma, with the activation of the MAPK and PI3K (phosphoinositide 3-kinase) pathways, which are responsible for cellular processes such as growth, proliferation, differentiation, and survival, leading to deregulation of the cell cycle and the inhibition of apoptosis, with pathways altered in 80 to 90% of melanomas [26]. Activation of the pathway is mainly due to mutation in the proto-oncogenes *BRAF*, *NRAS*, and *NF1* (neurofibromin 1). The BRAF protein has the function of phosphorylating MAPK/ERK kinase (MEK) activating it, and the NRAS protein also participates in the activation of the MEK and in the activation of the PI3K pathway. The NF1 protein has the function of regulation through the activation and inactivation of the RAS protein. In most melanomas, the mutation rarely occurs in BRAF and NRAS simultaneously, but when there is a mutation in any of these proteins, MEK activation occurs (MAPK pathway), and there is also the activation of the PI3K pathway, consequently, there will be an excess of expression of the inflammatory factors, growth factors, and the suppression of apoptotic factors [3,4,27]. The beginning of the transformation of melanocytes into melanoma mostly occurs through the mutation of these proteins, which consequently activates the cellular senescence [1]. In the development of melanoma, mutation occurs in the telomerase reverse transcriptase (*TERT*) gene, increasing its transcription. Telomerase has the function of protecting cells against apoptosis (suppressing caspase-mediated apoptosis), thus preventing cell senescence, and contributing to the growth and development of melanoma [1,3]. Another pathway that controls the transformation into melanoma is the microphthalmia-associated transcription factor (MITF) pathway, which is essential for the development of melanocytes, in addition to melanin synthesis, and is also responsible for the differentiation, growth, and survival of melanocytes [3,28]. When mutated, *MITF* increases its activity, leading to an increase in transcription factors such as *CDK2* (cyclin-dependent kinase 2), and CDK2 is responsible for the cell cycle regulation [3,27], and it was observed that the depletion of CDK2 suppressed the cell growth and cell cycle progression in melanoma [29].

The ability of melanoma to metastasize is mostly confirmed in two genes with mutations that regulate cell death and cell cycle, the *TP53* gene (that encodes the tumor protein p53, or p53) and retinoblastoma *RB* (that encodes the retinoblastoma protein, pRb), respectively [27,30]. Furthermore, alterations in the *CDKN2A* (cyclin-dependent kinase inhibitor 2A) gene, which encodes two proteins, p14 (ARF) and p16 ((INK4A), which inhibit CDK, are relevant regulators of cell cycle checkpoints. The protein p53 has an important role as a tumor suppressor, essential for regulating cell division and senescence, but its most relevant function is the ability to activate apoptosis. The protein p53 is protected by p14, as p14 allows for p53 induction in response to DNA damage and protects p53 from degradation [31]. The main role of pRb is to prevent excessive cell growth by inhibiting cell cycle progression until the cell is ready to divide; when the cell is ready to divide pRb is phosphorylated, thus being inactivated, allowing for progress of the cell cycle [32], and p16, by inhibiting CDKs, prevents pRb phosphorylation. Thus, the gene *CDKN2A*, under normal conditions, regulates p16, promoting cell cycle arrest by inhibiting pRb phosphorylation by CDK4, and regulates p14, which regulates the p53 protein through the interaction with MDM2 (negative regulator of p53). When there is a mutation in the *MDM2* (mouse double minute 2 homolog) gene, the proteins become dysfunctional, allowing the cell cycle to advance without control and the suppression of p53 occurs, with no apoptotic induced mechanisms [3,28,33]. These mechanisms allow for advancement in the development of cell growth, and consequently, the metastasis of melanoma.

## 3. Natural Compounds against Melanoma In Vitro

**Figure 2:**
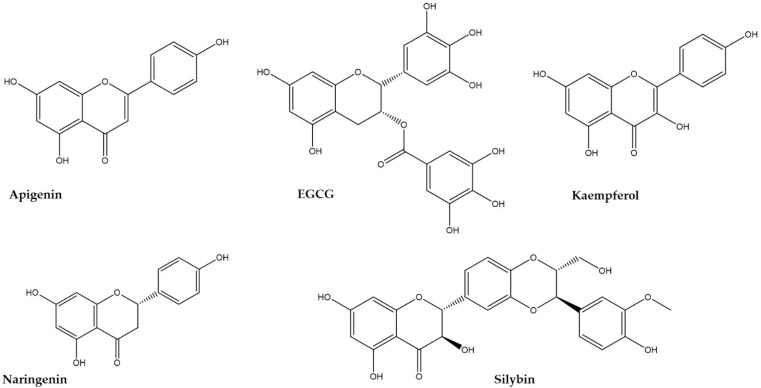
The chemical structure of the five flavonoids selected in this review, namely, apigenin (flavone), epigallocatechin-3-gallate (EGCG, catechin), kaempferol (flavonol), naringenin (flavanone), and silybin (flavonoid derivative).

Natural compounds such as polyphenols have been recognized as having important bioactivities including anti-mutagenic and anticancer properties due to their capacity to act as modulators of several cellular pathways involved in cell growth and proliferation to scavenge ROS that have deleterious effects on DNA, proteins, and lipids. However, they can also stimulate the repair of DNA, absorb UV-radiation, and have other positive effects [7]. Among the polyphenols, flavonoids that comprise a vast group of polyphenolic compounds with a characteristic benzo-γ-pyrone structure have important biological activities that are dependent on the degree of hydroxylation, substitutions, and conjugations of the backbone structure [12]. These compounds are easily available as nutrients in regular diets, and due to their prominent pharmaceutic potential shown in vitro and in vivo, many of these compounds are referred to as nutraceuticals [7,11,17]. In this review, five common flavonoids, namely, apigenin, epigallocatechin-3-gallate (EGCG), kaempferol, naringenin, and silybin were chosen (Figure 2), and relevant studies using these compounds as anti-tumoral agents as well as strategies to improve their bioavailability and efficacy were described.

### 3.1. Apigenin

**Figure 3:**
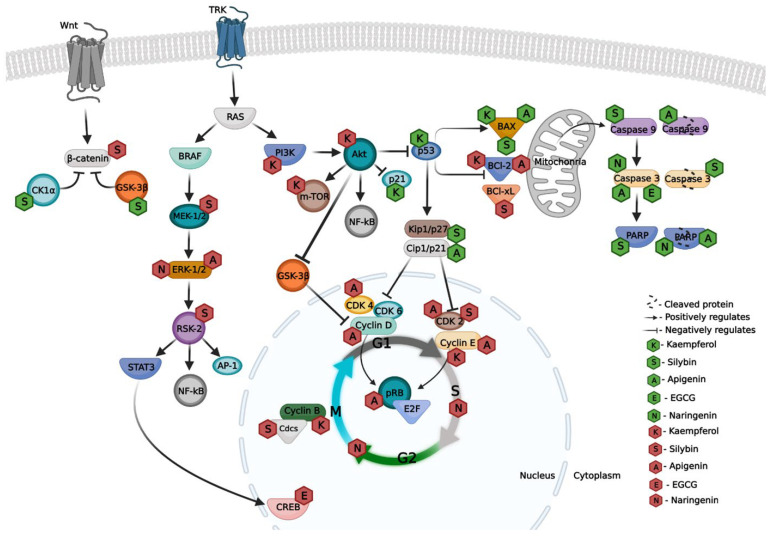
The Wnt and TRK signaling pathways as the main metabolic pathways mutated in melanoma and the respective site of action of the selected phenolic compounds. The inhibitory effect is denoted by red symbols and the protein activation is denoted by green symbols (see the legend at the right). Please see text for details. Abbreviations: K—Kaempferol; S—Silybin; A—Apigenin; E—Epigallocathechin-3-gallate (EGCG); CK1α—Casein kinase 1 α; GSK-3β—Glycogen synthase kinase-3 beta; RAS—Rat sarcoma virus; BRAF—Proto-oncogene B-Raf; MEK-1/2—Mitogen-activated protein kinase kinase 1/2; ERK-1/2—Extracellular signal-regulated kinase 1/2; RSK-2—Ribosomal s6 kinase 2; STAT3—Signal transducer and activator of transcription 3; NF-kB—Nuclear factor kappa-light-chain-enhancer of activated B cells; AP-1—Activator protein 1; PI3K—Phosphoinositide 3-kinase; Akt—Protein kinase B; m-TOR—Mammalian target of rapamycin; p21—Cyclin-dependent kinase inhibitor 1; p53—Cellular tumor antigen p53; BAX—BCL2 Associated X; BCl-2—B-cell lymphoma 2; BCl-xL—B-cell lymphoma-extra-large; PARP—Poly (ADP-ribose) polymerase; Kip1/p27—Cyclin-dependent kinase inhibitor 1B; CDK2/4/6—Cyclin dependent kinase 2/4/6; CREB—cAMP response element-binding protein; Cdcs—Cell division control proteins; pRB—Retinoblastoma protein; E2F—Transcription factor E2F; TRK—Tyrosine kinase receptor; G1/S/G2/M—Cell cycle phases.

Apigenin is a flavonoid (flavone class) that is present in several fruits, vegetables, and medicinal and aromatic plants that are used in traditional medicine, being attributed with several bioactivities such as anti-inflammatory, antioxidant, and anticancer, among others [12,15,34]. The anticancer activity was studied by Woo, et al. [15] through the exposure of the A375P and A375SM human melanoma cell lines to apigenin, having verified the inhibition of cell growth as a result of the activation of the apoptotic pathway, as explained by the raised levels of apoptotic proteins (BAX, p53, cleaved caspase 9, caspase 9, caspase 3, and cleaved PARP) and by a decrease in the anti-apoptotic protein BCl-2 [15,35]. In addition, apigenin induced time- and dose-dependent cytotoxicity to the melanoma A375 and A2058 cell lines accompanied by a reduction in the cell number and induced morphological changes into a rounded shape compatible with anoikis, which was mainly due to the reduction in the integrin protein levels [36]. These authors also showed that apigenin inhibited the phosphorylation of focal adhesion kinase (p-FAK) and extracellular signal-regulated kinase (ERK), thereby decreasing cell migration and consequently decreasing melanoma metastasis, and induced apoptosis by increasing the levels of caspase 3 and cleaved PARP (poly(ADP-ribose) polymerase) [36]. Using the human melanoma A375 and C8161 cell lines, apigenin up to 280 μM induced cell cycle arrest in the G2/M phase and apoptosis with the increase in cleaved caspase 3 [37]. Apigenin was also shown to target the expression of the Kip/p27 protein in the WM1361B and WM983A melanoma cell lines, positively regulating it, resulting in a decrease in cyclin-D and cyclin-E, and in CDK2/4/6 proteins, which did not allow for breakage of the binding between the RB and E2F proteins, thus having a cell cycle arrest in the G1/G0 phase [34]. These data show that apigenin has the ability to target relevant points in several signaling pathways of the melanoma cells (Figure 3, Table 1).

### 3.2. Epigallocatechin-3-Gallate

EGCG is one of the major flavonoids (catechin class) of the plant *Camellia sinensis* L., which has several physiological benefits such as anti-inflammatory, antioxidant, and anti-cancer activity, among others [38,39,40]. The activity of EGCG against melanoma was studied by Zhang, et al. [38], who found that exposure of the mouse melanoma cell line, B16F10, to EGCG (up to 100 μM) dose-dependently decreased the cell viability, and also decreased the levels of the MITF protein through the inhibition of CREB phosphorylation. As previously mentioned, the MITF protein is essential in melanocytes, but it is increased when the cell is cancerous, as it also controls the cell cycle [38]. In the human melanoma A375 cell line, EGCG inhibited growth through the activation of the apoptotic pathway, with an increase in the pro-apoptotic protein caspase 3 and with a decrease in the anti-apoptotic protein BCl-2. In this cell line, EGCG also acts in the AMPK pathway, decreasing the phosphorylation of this protein, indicating that there was a decrease in autophagy through this pathway as well as an increase in the PI3K/AKT/mTOR pathway proteins, thus showing that EGCG also has a negative regulation of autophagy via this pathway [16]. Thus, EGCG also acts on the relevant steps of the melanoma pathways (Figure 3, Table 1)

### 3.3. Kaempferol

Kaempferol is a flavonoid that is present in several vegetables, fruits, and plants, and has several physiological bioactivities such as anticancer, antibacterial, and anti-inflammatory activities, among others. At the cellular level, in cancer cells, kaempferol can regulate the cell cycle, activate apoptotic pathways, and in some cases, inhibit the migration and invasion of some cancer cells [41,42]. Kaempferol also interfered with mechanisms against the in vitro growth of melanoma cells. It promoted cell cycle arrest at the G1 phase in the A375SM cell line through the inhibition of cyclin-E and cyclin-B, mediated by the increased expression of p21, which downregulates the first two proteins. Kaempferol caused an intracellular increase in ROS, thus activating the mitochondrial apoptotic pathway, which allowed for a phosphorylation of p38 MAPK, and consequently to an increase in p53 (which activated p21), increasing the apoptotic protein BAX and decreasing the anti-apoptotic protein BCl-2, thus blocking cell division and allowing apoptosis to occur [13]. Yang, et al. [41] and Qiang, et al. [42] showed that kaempferol induced the cell cycle arrest of A375 and B16 cells in the G2/M phase. Yang, et al. [41] also showed that kaempferol caused, the in A375 cell line, a low regulation of the m-TOR, PI3K, and Akt proteins, thus inhibiting the m-TOR/PI3K/Akt pathway, which is an important pathway for the growth of melanoma. Thus, kaempferol also acts on the relevant steps of the melanoma pathways (Figure 3, Table 1).

### 3.4. Naringenin

Naringenin is a flavanone that is mostly present in citrus fruits such as orange, grapefruit, and mandarin. It has been described to have several bioactivities of interest such as anti-inflammatory, antioxidant, and antitumor properties [43,44]. Naringenin was reported to inhibit the proliferation and migration of melanoma cell lines SK-MEL-28 and B16F10. Naringenin acts on the phosphorylation of the ERK1/2 and JNK proteins, inhibiting it, thus inhibiting the activation of these proteins that are essential for the survival and proliferation of melanocytes. Furthermore, it also has the ability to activate apoptotic pathways through increasing caspase 3 expression, which consequently increases PARP cleavage. These proteins are essential for apoptosis, thus indicating that naringenin induces apoptosis in melanoma cells [43]. Nasr Bouzaiene, et al. [45] showed that naringenin induces cell cycle arrest at the subG1, S, and G2/M phase in the B16F10 cell line, not allowing the cells to multiply. At the cellular level, cell cycle arrest may induce DNA repair so that the cell can divide without mutations; if this repair does not occur properly, then the cell may induce apoptosis or necrosis [45]. Choi, et al. [43] reported that naringerin induces apoptosis, which may be the pathway that will be active after cell cycle arrest (Figure 3, Table 1). Naringenin is thus considered as another flavonoid of interest, with a documented ability to induce apoptosis through cell cycle arrest, and is potentially useful against the development of melanoma.

### 3.5. Silybin

Silybin is the main compound of *Silybum marianum* L. Gaertn. (*Carduus marianus* L. or milk thistle), which has several bioactivities such as anti-cancer, anti-inflammatory, antioxidant, and the prevention of allergies, among others [14,46]. In melanoma, silybin has anti-metastasis activity via the Wnt/β-catenin signaling pathway (Figure 3) in melanoma cell lines. β-catenin is a protein that has a high importance in cell-to-cell adhesion when present in the cell cytosol, not allowing cell migration; if it is active and goes to the cell nucleus, there will be an increase in the transcription and cell migration, thereby increasing the migration of cancer cells. Therefore, silybin increases the expression of GSK-3β and CK1, thus allowing for the phosphorylation of β-catenin, leading to its degradation. An increase in β-catenin in the cytosol and a decrease at the nuclear level was confirmed by the decrease in MMP-2/9 proteins, which are essential for the expression of β-catenin [47]. Vaid, et al. [14] showed that silybin promotes cell cycle arrest in melanoma cell lines. In the A375 cell line, silybin induced cell cycle arrest in the G0/G1 phase, as demonstrated by the decrease in cyclin D and CDK proteins, caused by the increase in the precursor inhibition proteins Cip1/p21 and Kip1/p27. However, in the Hs294T cell line, silybin caused a cell cycle arrest in the G2/M phase, which was confirmed by the decrease in cyclin-B expression and the consequent inhibition of the expression of the Cdcs precursor proteins. In both of these cell lines, and in addition to cell cycle arrest, silybin induced apoptosis, increasing the pro-apoptotic protein BAX and decreasing the anti-apoptotic proteins BCl-2 and BCl-x, further causing the activation of caspases 3 and 9 and the PARP protein, these being essential factors of apoptosis [14]. Furthermore, it is described that silybin is an inhibitor of the BRAF-MEK-ERK-RSK2 pathway, which is an important pathway for the growth, proliferation, and survival of melanoma. In order to prove this, Lee, et al. [46] exposed the SK-MEL-5 and 28 cell lines to silybin and showed that it had the ability to downregulate MEK1/2, ERK2, and RSK2 proteins, thus blocking the activity of AP-1, STAT3, and NF-kB, which are responsible for the development and progression of melanoma. Thus, silybin also acts on the relevant steps of the melanoma cell pathways (Figure 3, Table 1). 

## 4. Natural Compounds Function in Melanogenesis

Melanogenesis is a biochemical process that occurs in melanocytes, within melanosomes, involving several enzymes, with the production of a pigment called melanin being the main purpose. Melanin production is dependent on the oxidation of L-tyrosine to L-dopaquinone, which is mediated by the enzyme tyrosine (TYR), being limited by the hydroxylation of L-tyrosine to L-3,4-dihydroxyphenylalanine (L-DOPA). α-Melanocyte stimulating hormone (α-MSH) is also present in this process, positively regulating melanogenesis and stimulating the microphthalmia transcription factor (MITF), thus activating TYR. Melanin plays an important role in protecting against damage caused by ultraviolet radiation (UVR), however, a high stimulation of melanin production can lead to other skin diseases [11,48]. Some flavonoids have the ability to modulate melanogenesis through the action of these compounds at various stages of the pathway. For example, apigenin has been shown to have melanogenic activity, thus increasing melanogenesis, which has been verified through increased tyrosinase activity. Ye, et al. [49] also showed that exposure of the B16 cell line (mouse melanoma cell line) to apigenin increased the melanin content in these cells as well as an increase in the expression of TRP-1 and TRP-2, these enzymes being essential for the synthesis of melanin [49]. Ye, et al. [50] found that in the same cell line, apigenin increased the expression of MITF, which positively regulated the transcription of the TYR, TRP-1, and TRP-2 enzymes, thus corroborating the effect of apigenin on melanogenesis [50]. In contrast, EGCG showed the ability to inhibit the activity of the tyrosinase enzyme and to also decrease the melanin content in the B16F10 cell line (mouse melanoma cell line). Zhang, et al. [38], by exposing the B16F10 cell line to EGCG, quantified the expression of proteins that interacted with melanogenesis, with a decrease in the cAMP expression, which is responsible for activating the phosphorylation of the CREB transcription factor. The expression of this factor also decreased in the exposure of the cell line to EGCG; CREB has the function of activating the transcription factor MITF, which was also reduced under the same conditions. Consequently, the quantification of TYR, TRP-1, and TRP-2 was shown to be reduced in cells exposed to EGCG compared to the control, thus showing that EGCG regulates melanogenesis negatively [38]. Regarding kaempferol, there was an inhibition of tyrosinase enzyme activity and a decrease in the melanin content in the B16 cell line, thus kaempferol is a potential inhibitor of melanogenesis [51]. The same was not observed in mouse melanoma cells (B16F1) when exposed to silybin, with an increase in tyrosinase activity and an increase in the melanin content compared to the control. Silybin also promoted the increase in melanogenesis-promoting proteins such as TRP-1, TRP-2, MITF, and even p-CREB, thus proving the melanogenic activity induced by silybin in this cell line [52]. In addition to modulating these flavonoids, naringenin also has the ability to positively modulate melanogenesis. The exposure of B16F10 cells to naringenin promoted an increase in tyrosine activity and in melanin content as well as an increase in the MITF protein and its transcription factor β-catenin, thus promoting melanogenesis [44]. Flavonoids have the ability to modulate melanogenesis through several pathways and from several sites of the melanogenic metabolism.

## 5. Natural Compounds Function in Melanoma Prevention

As previously mentioned, one of the major causes of melanoma development is exposure to ultraviolet radiation (UVR), and the skin’s self-protection mechanisms are not sufficient for effective protection against UVR. The use of sunscreen is essential for this protection; however, it usually has chemical compounds that are toxic and/or cannot protect from the full range of radiation. Natural compounds such as flavonoids have the ability to absorb and protect the skin against UVR as well as having beneficial bioactivities for the skin such as antioxidant and anti-inflammatory activities, among others [53]. Apigenin showed the ability to prevent damage caused by ultraviolet radiation, and Britto, et al. [54] showed that apigenin had the ability to absorb ultraviolet radiation through the quantification of the sun protection factor, which had a value of 10.08, proving that it has the ability to protect from ultraviolet radiation [54]. In addition to preventing the formation of melanoma through UVR absorption, apigenin has the ability to prevent the growth of melanoma as shown by Zhao, et al. [37], where apigenin had the ability to inhibit the proliferation of skin melanoma cells (cell line A375 and C8161); after exposing these cells to apigenin for 24 h, an IC_50_ of approximately 100 µM was obtained for both cell lines. Zhao, et al. [37] was also able to prove a preventive effect of apigenin on metastasis (wound healing assay) and invasion (transwell chamber assay) of the same cell lines through its exposure to concentrations lower than the IC_50_ of apigenin; in these migration and invasion assays, it was found that a significant decrease number of cells migrated and passed the membrane compared to the control [37]. Through the wound healing assay on the 518A2 cell line (human melanoma cell line) exposed to apigenin, there was a significant inhibition of cell migration compared to the control group, corroborating the results above-mentioned. The action of apigenin on the actin cytoskeleton and on the β-catenin of this cell line was also evaluated; this one had a high metastasis potential, and a large part of the β-catenin was located in the cytoplasm and in the nucleus, which is typical of metastasis. In this cell line, apigenin showed a high disintegration of actin filaments as well as a difference in the positioning of β-catenin, showing itself mainly in the cell membrane, indicating that apigenin has a high anti-metastasizing power [55].

EGCG also showed a potential for UVR absorption, thus having the ability to protect from the sun. The SPF of EGCG was determined at a concentration of 400 µg/mL, obtaining a value of 31.02, thus showing a photoprotection classified as ultra-protection [56]. EGCG also showed anti-proliferative activity, and the study by [57] found that exposure of the B16F10 cell line for 24 h to EGCG inhibited cell proliferation, with an IC_50_ of 88 µM, not allowing melanoma to grow [57]. In addition to inhibiting the development of melanoma, EGCG has the potential to inhibit the migration of this type of cell. The B16F10 cell line was exposed to non-cytotoxic concentrations of EGCG and cell migration was assessed, where at 50 µM EGCG, about 50% of the cells did not migrate compared to the control group, showing a high anti-metastatic power on the part of EGCG [58]. There are several flavonoids with this type of protective activity against the formation and development of melanoma, another example is kaempferol, which has a high UVR absorption potential, showing an SPF value of 24.9, proving that it provides photoprotection against solar radiation [59]. This flavonoid has other bioactivities such as anti-proliferative and anti-migratory in melanoma cell lines. The A375 cell line (human melanoma cell line) was exposed to kaempferol for 48 h, yielding an IC_50_ of 20 µM. At this concentration, kaempferol also showed an ability to inhibit the migration of human melanoma cells when compared to the control, showing a preventive capacity in the formation, multiplication, and migration of melanoma [41]. The SPF of silybin was found to be 5.50, showing the ability to absorb some radiation from the sun, verifying that it presents photoprotection [60]. In addition to photoprotection, silybin showed the ability to reduce the viability of human melanoma cells (SK-MEL-5) in a concentration-dependent manner (10–80 µM) to about 50% viability after 24 h of exposure, showing an anti-proliferative power [46]. As previously described, silybin has the ability to inhibit β-catenin, which plays a fundamental role in cell invasion when it is in the nucleus and in cell-to-cell adhesion when it is in the cytoplasm. When its transport to the nucleus is inhibited by the cell, it loses the ability to migrate, showing that silybin has an anti-metastasizing power [47]. The photoprotective power of naringenin was tested, proving that the use of this flavonoid in the formulation increased the SPF from 6 to 8.4, showing some ability to absorb UV radiation and protect against solar radiation [61]. The antiproliferative and antimigratory power of naringenin was evaluated by Choi et al. [43] in two cell lines (B16F10 and SK-MEL-28) with a decrease in viability up to about 60% in both cell lines compared to the control. In cell migration, there was also a significant reduction of about 96.7% in the B16F10 cell line and 50.8% in the SK-MEL-28 cell line compared to the control, verifying that this flavonoid, in addition to its antiproliferative power, also has the ability to inhibit the migration of these melanoma cell lines [43].

## 6. Nanoengineering Delivery Systems for the Selected Flavonoids

Flavonoids as well as many natural molecules are very unstable in aqueous environment or present very low solubility, thus nanotechnology has arisen as a novel approach to encapsulate these molecules, protect them from degradations, and enhance their solubility and bioavailability, thus enhancing their activity as anticancer agents [7,62,63].

The topical application of flavonoids is the ideal approach to allow these bioactives to reach the melanocytes that are located beneath the keratinocytes, however, there are several biological and chemical barriers to overcome. For compounds to reach the target site, they must be absorbed by keratinocytes without changing their properties. One way that has been proposed to ensure that the activity of the compounds is kept at the target site is the encapsulation of these compounds into nanoparticles. The selection of the type of nanoparticle is greatly dependent on the physicochemical properties of the bioactives, in order to ensure a high bioavailability [64]. Chao et al. [65] showed that loading kaempferol in submicron-sized emulsions increased the amount of flavonoid that was deposited on the skin, thereby promoting a faster absorption through the skin [65]. Silybin showed several bioactivities when applied topically to rats, but when loaded into solid lipid nanoparticles, the deposition of silybin on the skin increased, also increasing its topical absorption [66]. The solubility of silybin was increased when formulated in a microemulsion and in dendrimers, which improved its chemical and physical stabilization, and allowed for a prolonged release profile of this flavonoid [67]. The anticancer activity of apigenin was increased when loaded into PLGA nanoparticles [68], which also promoted the physicochemical stability of this bioactive [35]. Ceramide-based nanostructured lipid carriers were proposed to increase the loading capacity of apigenin, thus contributing to an increase in its permeability in the skin [69]. The chemical stability of apigenin and its bioactivity were also improved by its loading into nanoemulsions and transferosomes, where both systems also contributed to modify its release profile [70,71]. EGCG has the same absorption and stability problems as other flavonoids, and has also been proposed to be loaded into different types of nanoparticles to improve its bioavailability [72,73]. Chamcheu, et al. [74] and Shetty, et al. [75] encapsulated EGCG in cationic nanoparticles and dendrimers, respectively, which significantly improved the flavonoid activity, stability, and bioavailability [74,75]. The same was reported for the encapsulation of EGCG into nanoethosomes and transfersomes, with a significant increase in the activity of EGCG against melanoma [76,77]. Functionalized multiwalled carbon nanotubes were proposed to increase the bioactivity of naringenin, and their cytotoxicity was evaluated against the human fibroblasts cell line, showing lower cytotoxicity in non-malignant cells than the free compound [78]. A summary of the nanosystems used to encapsulate the selected flavonoids is presented in Table 2.

## 7. Conclusions

Melanoma is a complex type of cancer that depends on several metabolic factors, and the currently used therapies are not always effective and have unwanted side effects. In this review, we report that phenolic compounds have a high potential to act against melanoma in several key points of its developmental stages, and also reduced the risk of metastasis. However, phenolic compounds have some difficulty in reaching the target site, and with the encapsulation of these compounds into nanoparticles, it is possible to achieve greater stabilization of the compounds, greater topical absorption, and greater bioavailability. Phenolic compounds have a high potential against melanoma and can be recommended for use in the therapy or mitigation of melanoma and its invasion and metastasis. Furthermore, these compounds may be used as co-adjuvants of the current therapies due to their antioxidant and anti-inflammatory activities.

## Figures and Tables

**Table 1:** A summary of the key proteins and genes that are targets to the selected flavonoid compounds. The effect of flavonoids on the cell cycle phases is also highlighted.

Compound	Melanoma Cell Line	Effects	Ref.
Apigenin	A375P and A375SM	↑ BAX ↑ p53 ↑ Cleaved caspase-9 ↑ Cleaved PARP ↓ BCl-2	[15]
	A375 and A2058	↓ p-FAK ↓ p-ERK-1/2 ↑ Caspase-3 ↑ Cleaved PARP	[36]
	A375 and C8161	↑ G2/M (cell cycle) ↑ Cleaved caspase 3	[37]
	A375	↑ Caspase 3 ↑ Caspase 9 ↑ BAX ↓ BCl-2	[35]
	WM1361B and WM983A	↑ G0/G1 (cell cycle) ↓ Cyclin D1/2 ↓ Cyclin E ↓ CDK2/4/6 ↑ p27Kip1 ↓ pRB ↓ E2F	[34]
EGCG	B16F10	↓ p-CREB ↓ CREB ↓ MITF	[38]
	A375	↑ Caspase 3 ↓ BCl-2 ↑ p-PI3K ↑ p-AKT ↑ p-mTOR ↓ p-AMPK	[16]
Kaempferol	A375SM	↑ p21 ↓ Cyclin E and B ↑ p38 MAPK ↑ p53 ↓ BCl-2 ↑ BAX	[13]
	A375	↑ G2/M (cell cycle) ↓ m-TOR↓ PI3K↓ Akt	[41]
	B16	↑ G2/M (cell cycle)	[42]
Naringenin	B16F10 and SK-MEL-28	↓ p-ERK1/2 ↓ p-JNK ↑ Caspase 3 ↑ Cleaved PARP	[43]
	B16F10	↑ subG0/G1; ↑ S; ↑ G2/M (cell cycle) ↓ G0/G1 (cell cycle)	[45]
Silybin	A375 and HS294T	↓ Nuclear β-catenin ↑ Cytosolic β-catenin ↓ MMP-2 and MMP-9 ↑ p-β-catenin ↑ CK1α ↑ GSK-3β ↓ BCl-2 and BCl-x ↑ BAX ↑ Caspase 9 ↑ Cleaved Caspase 3 ↑ PARP	[14,47]

Note: the changes are denoted by upward arrow (↑) meaning increase and downward arrow (↓) meaning decrease.

**Table 2:** The nanoparticles of different composition that have already been studied and proposed to encapsulate and deliver the selected flavonoids. The in vitro models where these nanoparticles were effective in delivering the indicated flavonoid are also indicated.

Compound	Nanoparticle	In Vitro Model	Ref.
Apigenin	PLGA nanoparticles	Rat skin Cell culture	[35,68]
	Nanostructured lipid carriers	Pig skin	[69]
	Nanoemulsion	Artificial skin	[70]
EGCG	Cationic nanoparticles	Rat skin	[74]
	Nanoethosomes	Rat skin	[76]
	Dendrimer	Rat skin	[75]
	Transferosomes	Rat skin	[77]
Kaempferol	Submicron emulsion	Rat skin	[65]
Naringenin	Carbon nanotubes	Fibroblast cell line	[78]
Silybin	Solid lipid nanoparticles	Rat skin	[66]
	Microemulsion	Pig skin	[67]
	Dendrimer	Rat skin	[75]

## Data Availability

Not applicable.
